# Correction to “Conservation applications of niche modeling: Native and naturalized ferns may compete for limited Hawaiian dryland habitat”

**DOI:** 10.1002/aps3.70052

**Published:** 2026-03-25

Edwards‐Calma, K., L. Jiménez, R. Zenil‐Ferguson, K. Heyduk, M. K. Thomas, and C. M. Tribble. 2024. Conservation applications of niche modeling: Native and naturalized ferns may compete for limited Hawaiian dryland habitat. *Applications in Plant Sciences* 12(3): e11598. https://doi.org/10.1002/aps3.11598

The last sentence of the original Data Availability Statement read: “The rasters with the climate variables and land‐use layers can be found at their original sources using the links provided in the Methods section.” The corrected sentence reads: “The rasters with the climate variables and land‐use layers can be found on Zenodo: doi.org/10.5281/zenodo.18985966.”

We apologize for this error.

